# Hype vs. Health: How Approved Nanomedicines Have Met (or Missed) Early Predictions

**DOI:** 10.3390/nano16050284

**Published:** 2026-02-24

**Authors:** Eleonore Fröhlich

**Affiliations:** 1 Center for Medical Research, Medical University of Graz, Stiftingtalstr. 24, 8010 Graz, Austria; eleonore.froehlich@medunigraz.at; 2 Research Center Pharmaceutical Engineering GmbH, 8010 Graz, Austria

**Keywords:** nanodrugs, nano-enabled devices, approved nanomedicines, in silico tools, nanotoxicology

## Abstract

Two decades after the first bold proclamations that nanomedicine would deliver “magic-bullet” therapies capable of cell-level targeting, the field stands at a crossroads. While some initial promises (improved delivery of poorly water-soluble drugs and enhanced efficacy and biocompatibility of nano-based devices) have been fulfilled, other early promises (active targeting, biodegradability, multifunctionality, triggered responses, real-time data output, and implantable sensors) remain only partially realized. This article will compare the properties of approved nano-based products to those of the ideal products, assess the shortcomings of existing nano-based products, and discuss critical issues in nanotoxicity (biodistribution and protein corona effects, immune interactions, and biopersistence) and the lack of data on product and end-of-life life cycle analyses. The role of in silico tools in the various steps of nanodrug and nano-based device development and manufacturing—areas in which these tools are the most established (nanocarrier design, prediction of cellular effects, chemical composition optimization, manufacturing, and signal interpretation)—is also addressed. Future goals include biodegradable targeted delivery systems, better tissue integration of implants, and implantable sensors. It is expected that, alongside careful physicochemical characterization of the nanoproduct, toxicity testing focused on nano-specific effects and life cycle analyses of production and end-of-life phases will facilitate the approval of nano-based products.

## 1. Introduction

The term “nanotechnology” was coined in 1974 by Norio Taniguchi to describe fabrication and control of matter at the atomic scale. K. Eric Drexler later popularized medical applications of nanotechnology in *Engines of Creation* (1986) [1]. Journals dedicated to nanoparticle research emerged in the 1990s (e.g., *Advanced Materials*) and expanded substantially in the early 2000s (e.g., *Nano Letters*, *Nature Nanotechnology*, *International Journal of Nanoscience, Journal of Nanobiotechnology*, *Nanotechnology*, *Science and Applications*, *Nanomaterials*), driven largely by academic work on medical nanoparticles [2]. The 15th anniversary of *Nanomaterials* provides a timely opportunity to compare early expectations for medical nanomaterials with products that have ultimately been approved.

Nanomedicines are medical products specifically formulated using nanotechnology tools and/or nanoscale materials for the prevention or treatment of human disease. They include nanodrugs and nano-enabled medical devices. A nanodrug can be defined as a therapeutic product in which nanotechnology is an integral part of the medicinal formulation or delivery system, and whose nanoscale properties materially affect the drug’s behavior (pharmacokinetics, biodistribution, stability, cellular uptake, or release). A nano-enabled device is a medical device whose function, performance, safety, or usability relies on engineered nanomaterials or nanoscale structures. The device may be diagnostic, therapeutic, or supportive. The types of nanoparticles used in devices typically differ from those used in drug products; drug formulations generally employ soft, biodegradable particles, whereas nano-enabled medical devices often contain non-biodegradable materials such as silver, gold, iron oxide, titanium, and hydroxyapatite [3].

The following text intends to provide a general overview of the development of nanoparticle-based medical products, including nanotoxicity and life cycle analysis (LCA), over the last 10-20 years. Individual underlying mechanisms are not discussed in detail.

## 2. Dreams and Reality in Nanomedicine

**Figure 1:**
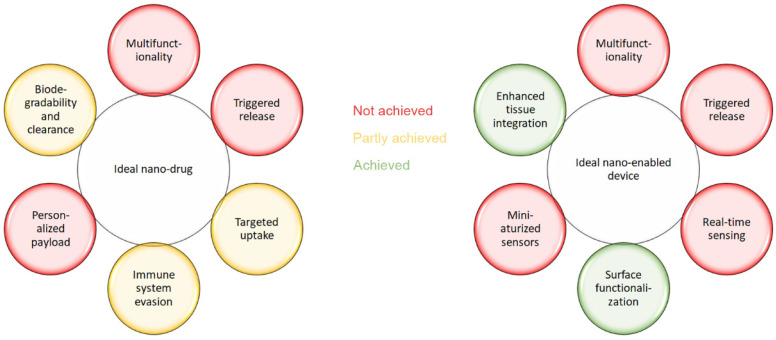
Color-coded parameters of ideal nanoproducts that were achieved (green), partially achieved (yellow), or not achieved (red).

Early visions of nanoparticles in medicine imagined autonomous nanorobots patrolling the bloodstream, machines that repair DNA or mitochondria, in-body nanofactories synthesizing drugs on demand, nanoscale mechanical actuators, Global Positioning System (GPS) tracking of individual particles, or targeted uptake of theranostic particles for personalized treatment and recording of therapeutic efficacy [4]. Reality has followed a different path. Figure 1 compares early expectations with Food and Drug Administration (FDA)-approved nanomedicines and shows that some goals, e.g., triggered release and multifunctionality, could not be realized for both nanodrugs and nano-enabled devices.

### 2.1. Nanodrugs

Expectations for nanodrugs included longer circulation times, stabilization of labile drugs, improved targeting, reduced off-target toxicity, and controlled release. Although the protection of labile molecules and delivery of higher payloads with lower systemic toxicity were achieved, other goals proved to be more difficult than anticipated. Table 1 compares the features of ideal and FDA-approved products.

Passive targeting via the enhanced permeability and retention (EPR) effect was thought to be sufficient for tumor targeting. In practice, EPR is highly variable between patients; reticuloendothelial system (RES) clearance is high, and tumor accumulation is often poor [5]. Active targeting (“magic bullets”) also underperformed in the clinic because of protein corona formation, heterogeneity of tumor markers, and limited in vivo targeting efficacy [6]. Stimuli-responsive “smart” nanoparticles are under development but face challenges from complex designs that can reduce in vivo stability and complicate regulatory approval [7].

The differences between idealized concepts and FDA-approved products are due to biological and pharmaceutical constraints. Access to organs—or to individual cells within tissues—is restricted by particle size, whereby particles may be too large to reach the intended target [8]. Furthermore, production proved more complex than anticipated. The requirements for reproducible purity, identity, and potency were more difficult to meet for nanodrugs, and the increased manufacturing complexity made scaling up more challenging and more costly. It also turned out that stability and shelf life are more critical for nanodrugs than for conventional drugs. Demonstrating product reproducibility, homogeneity, and stability created challenges for regulatory agencies reviewing such applications and issuing specific guidelines [9]. Drug release and carrier degradation proved to be less predictable than for conventional drugs. This is because nanoparticles adsorb macromolecules (e.g., protein corona), which can mask and modify release behavior [10]. Furthermore, release may occur at different locations—extracellularly or intracellularly (endosomes, lysosomes, cytosol). Tracking nanocarrier degradation is also difficult because multiple mechanisms are involved and the biological environment (pH, enzymes, reactive oxygen species (ROS) levels, protein corona) influences outcomes [11]. Small changes in formulation can have pronounced effects on nanocarrier degradation. Unanticipated immune responses, such as complement activation-related pseudoallergy (CARPA), present toxicological challenges. Moreover, the long-term toxicity of many inorganic nanoparticles remains uncertain [12]. High costs and only modest advantages over conventional therapies have further slowed the broad replacement of traditional drugs by nanodrugs [13]. Finally, clinical trial design—including patient selection and biomarkers—has had to be adapted for nanodrugs.

Although the originally envisioned multifunctional nanodrugs are not widely available, key goals have been achieved: reduced toxicity, fewer off-target effects, and increased stability and bioavailability. Although theranostic nanoparticles are not available, ^99m^Tc-labeled sulfur colloid, albumin colloid, SnF_2_ and Re_2_S_7_ colloids, and iron oxide nanoparticles are used in clinical practice [14]. Clearance of nanodrugs by the RES was reduced by functionalization with polyethylene glycol (PEG).

### 2.2. Nano-Enabled Devices

Physical and biological constraints make the original vision of autonomous in vivo nanorobots impractical today. Key obstacles include biocompatibility (e.g., thrombosis risk), immunogenicity [15], propulsion limitations (chemical fuels such as H_2_O_2_ are unsuitable in vivo), and complex navigation in viscous fluids and tortuous vessels. Removal from the body (retrieval, biodegradation, or excretion) and good manufacturing practice (GMP)-compliant manufacture of billions of tiny devices with consistent performances are also major challenges [16].

From the production side, predominantly, insufficient stability against sterilization and cleaning poses problems that contribute to the failure of nano-enabled devices in clinical practice [17]. A particular issue for nano-enabled devices may be the nanowaste generated during mass production of medical devices [18]. Toxicity issues may arise when nanoparticles leaching from devices enter surrounding tissue or the systemic circulation. This is relevant for nanosensors, which may contain carbon-based nanoparticles such as graphene and carbon dots, whose cytotoxicity is less well established than that of nanocarriers [19].

Regulatory classification (drug/device/biologic/combination), trial design, informed consent, privacy (if sensing), and environmental release concerns further complicate translation.

An example of the unclear situation are iron oxide nanoparticles, which can be classified either as a “nanodrug” or as a “nano-enabled device.” When injected for imaging, they are regulated as nanodrugs; however, if they are used as an antimicrobial coating, they would be treated as a device.

Nevertheless, progress has been made: adding nanoparticles to medical devices has improved biocompatibility, reduced implant-associated infections, enhanced surface properties, and enabled capabilities such as targeted drug release and biosensing [20].

## 3. Marketed Nano-Enabled Products

Despite intense research activity, relatively few nanoparticle-containing products are on the market. Most approved nanodrugs are in oncology: among drugs approved for cancer from 1998 to 2022, 15 of 198 were nanodrugs [21]. These are mainly liposomal formulations, both PEGylated and non-PEGylated; inorganic and polymeric nanoparticles are less common [22]. Nanodrugs do not fail at markedly higher rates overall compared with conventional drugs: the phase-1-to-approval success rate in oncology nanomedicine was 7% (2008–2020) [23], compared with a 3.4% overall approval likelihood for conventional drugs (2000–2015), and 11.4% for the lead indication [24]. However, causes of failure differ: nanodrugs most often fail for lack of efficacy, whereas conventional drugs commonly fail because of off-target toxicity or a lack of effect [25]. Regulatory approval of nanodrugs is often more complex because manufacturing requires extensive characterization (size, polydispersity, surface chemistry, encapsulation efficiency, release kinetics, stability) and detailed absorption, distribution, metabolism and excretion (ADME) testing due to altered circulation times and immunogenicity risks [26].

Although lessons from one nanoparticle class do not always transfer to others, general design principles for optimal nanoparticles can be illustrated by nab-paclitaxel (Abraxane) [9]: (i) a narrow size distribution in a specific range (~130 nm) avoids rapid renal and mononuclear phagocyte system (MPS) clearance; (ii) near-neutral or slightly negative surface charge minimizes aggregation; (iii) non-covalent payload binding enables drug release; and (iv) amorphous drug incorporation increases apparent solubility. Abraxane underwent extensive process optimization to ensure reproducibility. Yet, since Abraxane’s approval in 2005, no second-generation albumin or protein nanoparticles have been widely approved. Protein nanocarriers face additional translation challenges—batch variability, sensitivity of protein conformation to pH/temperature/ionic strength, potential immunogenicity (even from human proteins), purification difficulties for recombinant proteins, instability during sterilization, and costly cold-chain storage. 

Approved nano-enabled devices are mainly surface coatings and sensors. Nanotechnology has improved antibacterial properties, cellular responses, and electrical interfaces, particularly for local applications such as wound dressings and implants. In contrast to nanodrugs, there was no single “first uses”; rather, nanotechnology has driven gradual improvements to existing devices, and this process is still ongoing [27]. Device approval is often easier than drug approval because local action reduces systemic exposure, and device safety is guided by standardized tests (e.g., ISO 10993) and established biocompatibility endpoints [28]. Manufacturing and characterization are thus often less complex than for nanodrugs.

## 4. Types of Nanoparticles in Medicine

Recent reviews differ in the count of approved products, dependent on search methods, in the database clinicaltrials.gov. One review categorized 19 lipid-based, 17 polymeric + 2 protein-based, 15 nanocrystals, and 10 inorganic nanoparticles [29]; another listed 27 lipid-based, 27 polymeric, 25 nanocrystals, and 12 inorganic nanoparticles [30]. By 2025, roughly 91 approved nano-based products were on the market, compared to 1099 drugs approved by the FDA since 1995 (the year of the first approved nanodrug) [31,32].

**Figure 2:**
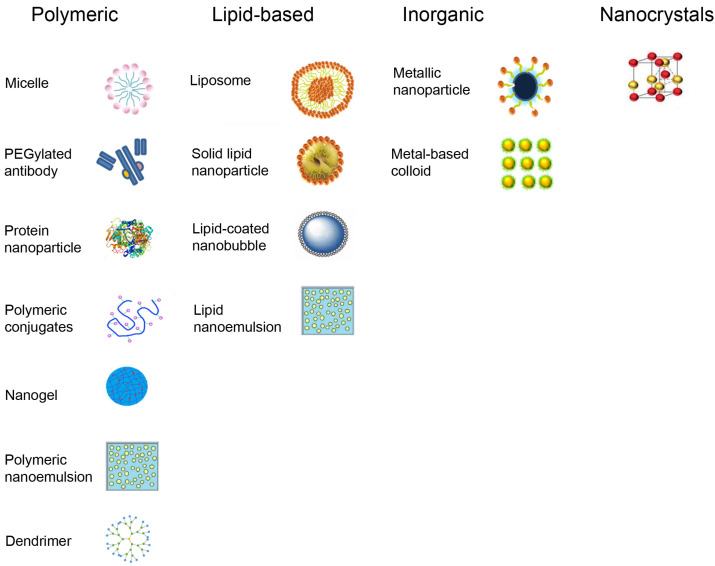
Types of nanoparticles found in approved nanodrugs and in nano-enabled medical devices.

Approved nanodrugs comprises protein nanoparticles, polymeric conjugates, polymeric micelles, PEGylated formulations, polymeric nanogels, copolymeric nanospheres, and nanoemulsions (Figure 2). Inorganic nanoparticles include iron-based agents, hafnium oxide, silver, and metal-based colloids for imaging. Nanocrystals have been made from diverse compounds (fenofibrate, dantrolene sodium, methylphenidate hydrochloride, naproxen sodium, paliperidone palmitate, cabotegravir, and others).

FDA-approved nano-enabled devices contain silver, silica, zirconia, titanium, and hydroxyapatite nanoparticles [20] and are used in orthopedics, cardiovascular, and neurological devices. Ex vivo nanosensors generally contain gold nanoparticles.

## 5. Administration Routes

In 2020, most approved nanodrugs were administered by injection (IV 44%, IM 8%, SC 15%), with only ~15% oral [33]. By contrast, small-molecule drugs were predominantly oral (~60% in 2016) [34]. Similar data were reported for 2017 with 62% of drugs administered in oral form and 22% by injection, 8.7% cutaneous, 5% mucosal (buccal, sublingual, rectal, vaginal, ocular), and 1% by inhalation [35]. The main indication was in oncology, where IV administration is common; 61% of medications in the year 2020 were IV [36]. Oral nanodrugs are increasing due to patient convenience, and lipid-based carriers are adaptable to various routes (e.g., pulmonary Arikayce, ocular nanomicelles and nanoemulsions such as Cequa and Restasis, dermal nanoemulsions and nadosomes such as Ameluz) [37]. Lipid emulsions for oral use (e.g., Neoral) are not labeled as nanodrugs because they form colloids in the gut, rather than delivering preformed, stable nanoparticles to the systemic circulation. Nanocrystals show promise for oral use and IV administration because of favorable dissolution, lack of harsh solvents in manufacture, and manageable clearance; approved oral nanocrystal products include Rapamune, Emend, and Megace ES [38].

## 6. Key Achievements in Nanomedicine

### 6.1. Nanodrugs

Initially dominated by lipid-based carriers for small molecules, the range of clinically available nanoparticles now includes also biologic payloads [39,40]. Table 2 lists drugs, their descriptions, and indications, including also antibody conjugates that not all researchers have classified as nanodrugs [41].

Nanodrugs, which have either lead to the development of more efficient products or advanced the understanding in the action of nanomedicines [30,42,43]:

- Adagen: first PEG-conjugated biologic providing reduced immunogenicity [44].
- Doxil (PEGylated liposomal doxorubicin): a first demonstration of stable encapsulation and improved tolerability [45].
- AmBisome: an amphotericin B liposomal formulation emphasizing stability and sterility [46].
- Mylotarg: increased efficacy by active targeting [47].
- Rapamune: improved oral bioavailability of the drug [48].
- Zevalin: improved radiation efficacy by active targeting [49].
- Abraxane: a solvent-free, albumin-bound formulation of paclitaxel [9,50].
- Ferumoxytol: first inorganic nanoparticle highlighting clearance and hypersensitivity concerns [51].
- Vyxeos: the first liposomal co-delivery formulation requiring controlled co-encapsulation ratios [42].
- Onpattro: the first clinical nanoparticle delivery of siRNA, underscoring biologic integrity and immunotoxicity concerns [42].
- mRNA lipid nanoparticle vaccines for coronavirus disease 2019 (COVID-19): demonstrated large scale GMP production, cold chain logistics, sequence integrity testing, and control of lipid impurities [42].
- Cabenuva: long-acting i.m. injectable product [52].

### 6.2. Nano-Enabled Devices

Nanosilver has been pivotal in antibacterial coatings for wound dressings (e.g., Acticoat), and in some dental and orthopedic products (e.g., Aquacel). Demonstrating controlled local release at efficacious, non-cytotoxic levels was key to approval [53]. Implants required additional focus on osseointegration and mechanical stability, and indwelling catheter coatings had to satisfy hemocompatibility requirements [54]. Silver coatings have been used for endotracheal tubes, intravenous catheters, urinary catheters, and surgical meshes; several products exist in these categories.

In addition to the first products of each kind as listed in Table 2, numerous products are available on the market as orthopedic, dental, and cardiovascular implants. They possess nanostructured surfaces and consist of titanium (Adaptix Interbody System, CON-DUIT, EIT Cellular Titanium), hydroxyapatite (Curiteva Porous PEEK Cervical Interbody Fusion System, Nanotite Dental Implants), silica and zirconia (Filtek Universal Restorative), ceramic nanohybrid resin composites (Pac-Dent Ceramic Nanohybrid Resin, Renamel NANO Plus), and polymer (COBRA PzF NanoCoated Coronary Stent System). More products are listed by Ma et al. [55]. The paclitaxel-coated balloon catheter uses drug-loaded nanoparticles (paclitaxel) on a device acting as a localized therapeutic delivery system [56].

Nano-enabled devices, which lead to the significant improvement of existing products:

- Colloidal gold-based test systems: markedly increased sensitivity [57].
- Silver-containing wound dressings: improved efficacy in wound and infection healing [53].
- Silver-coating of implants: improved antimicrobial effect [54].
- nanoLOCK: better osteogenesis [58].
- Gold or magnetic nanoparticle-based sensors: improved sensing [59].

## 7. Nanotoxicity

Toxicity mechanisms of nanodrugs and nano-enabled devices differ from conventional medical products (Table 3). Toxicity testing of nano-based products is more extensive because particle parameters—e.g., size, shape, surface area and functionalization, aggregation state, crystallinity, and porosity—impact biological effects. At the cellular level, this includes generation of ROS, release of metal ions (Ag^+^, Zn^2+^, and Cu^2+^), phagocytosis by phagocytes, and modulation of immune cell phenotypes [60]. Genotoxicity testing should be performed at an early stage because ROS and inflammation are known inducers of genotoxicity [61]. Since most nanoparticles are taken up by endocytosis, lysosomal dysfunction and lysosomal damage may occur.

At the organ level, specific biodistribution (EPR, RES accumulation), metabolization by ion release and oxidation, and decreased elimination (persistence after drug release, reduced renal clearance) are major causes of toxicity [62]. Marked effects are seen on the immune system because almost all immune cells are affected by nanoparticles [63]. Although some of these effects can be exploited for antitumor therapy, they are unwanted for other uses. These effects are mostly pro-inflammatory, for instance via the polarization of macrophages toward the M1 phenotype. Nanoparticles can also enhance antigen-presenting activity in dendritic cells, stimulate neutrophil degranulation, and increase or decrease T cell responses and natural killer cell activity. While initial effects are mainly pro-inflammatory, chronic nanoparticle exposure is more likely to induce myeloid-derived suppressor cells and an immunosuppressive milieu. PEGylation was initially believed to be the ideal coating to prevent nanoparticle uptake by phagocytes. However, it was shown that PEGylated liposomes can be subject to accelerated blood clearance through anti-PEG antibody formation or may induce hypersensitivity reactions by activation of the complement system, resulting in CARPA [64]. This finding has clinical relevance because anti-PEG antibodies were detected in 72% of human samples collected after 1999 [65]. Reproductive toxicity may occur due to oxidative stress, translocation of nanoparticles across placental barriers, and accumulation in reproductive organs [66]. After release from a device, nanoparticles show similar behavior to nanodrugs [67]. Dose calculation in this situation is difficult because high local doses and low systemic doses may occur. Release is the most likely during cleaning, sterilization, and disposal of nano-enabled devices.

The majority of in vitro and in vivo studies for nano-based products do not differ fundamentally from the testing of conventional drugs and devices [68]. However, several parameters are nano-specific and additional tests are suggested. These include dissolution to assess ion release, determination of the protein corona, intracellular localization of the particles, assessment of lysosomal function, inflammasome activation, phagocytosis testing, complement activation, neutrophil extracellular traps, and in vitro barrier translocation. In testing nano-enabled devices, the established guidelines for conventional devices can be used, but increased focus should be placed on particle/ion release, thrombocyte activation, and chronic inflammation. For assessment of biodistribution, biopersistence, and CARPA, in vivo experiments are needed. They should include markers for accumulation and chronic inflammation (e.g., study of RES organs, fibrosis, and granuloma formation). Specific analysis of inhalation exposure includes particle detection in BAL, neutrophil influx, and lung fibrosis.

Furthermore, interference with conventional assays may require adaptations. Exposure in physiological (protein-containing) solutions is recommended because the coating will influence cellular uptake and immune responses by opsonization. Conventional assays (e.g., for viability, immunoassays, ROS detection, lysosome function, etc.) should include controls for interference from light scattering and absorption, dye metabolism, and fluorescence quenching [69]. Advanced 3D cultures to assess permeation of epithelial barriers and induction of inflammation are recommended [70]. Testing for nanodrugs should determine carrier, unformulated drug, and formulation.

## 8. Life Cycle Assessment (LCA)

LCA is regulated in the ISO 14040/44 framework and includes (i) goal and scope, (ii) life cycle inventory, (iii) life cycle impact assessment, and (iv) life cycle interpretation [71]. The life cycle includes resources, production, utilization, disposal, and residues of nano-based products. Material discharged into the technosphere (wastewater treatment, waste incineration, recycling systems), the environment (water, soil, air), and living organisms (humans, animals, plants, fungi, microorganisms) are considered [72]. Thus far, LCA of nanomaterials in medicine has not been extensively studied, and LCA has been reported for only a few products (e.g., [73,74]).

For similar reasons as for toxicity, specialized approaches to traditional assessment methods are needed [75]. Whereas conventional materials are defined by chemical composition, for particles, size, shape, agglomeration, dissolved ions, and metabolization during production, use, sterilization, and disposal must be taken into account for nanomaterials [76]. Transformation of nanoparticles (e.g., aggregation, sedimentation, corona formation, surface changes) is more variable than biodegradation and volatilization of chemicals, and toxicity mechanisms, as mentioned in Section 7, are more complex.

Determination of the functional unit in nano-based medical products is difficult [77]. This was identified as a critical factor because in the LCAs concept, all inputs, outputs, and impacts are normalized to this parameter [71]. Since mass alone does not correlate with effects, other metrics such as particle number, total surface area, and reactive surface area should be indicated. Insufficient data on nanomaterial releases and transformations, high uncertainties in experimental measurements, and limited understanding of toxicity and exposure pathways have been identified as major limitations of LCA for nanomaterials in general. The life cycle includes production, utilization, and disposal, with the utilization stage having received the most attention in nanomedicine. Production of nanoparticles should also be considered because it can be highly energy consuming and lead to occupational exposure [78]. Further, environmental issues may arise from a lack of recycling or re-use. The use of green chemistry can positively impact sustainability and the environment. LCAs should be performed for every product separately, but there are specific aspects that differ between nanodrugs and nano-enabled devices (Table 4).

## 9. Future Trends

### 9.1. Nano-Based Products

Ongoing and emerging clinical trials indicate future directions: more complex functionalization and loading of nanodrugs, receptor-targeted liposomes for the transferrin receptor, prostate-specific membrane antigen (PSMA), human epidermal growth factor 2 (HER2), and intracellularly targeted gold nanoparticle spherical nucleic acids (siRNA; NCT03020017). Functionalization remains limited because size, shape, and surface properties are tightly linked to biological behavior. Optimal size is fixed between 50 and 250 nm based on the finding that clearance by RES is most effective for non-PEGylated liposomes <50 nm and >250 nm [79]. Shape influences biological responses—for example, Doxil hypersensitivity was linked to ovoidal rather than spheroidal liposome shapes [80]. Surface PEGylation strongly affects circulation time (e.g., Myocet ~2.5 h vs. Doxil ~55 h) [81]. Increasing design complexity raises the risk of unpredictable interactions. Delivery across the blood–brain barrier remains an active research area; liposomes and exosome-coated particles show promise [82]. Personalized nanoparticle treatments with adaptable payloads are an emerging trend, but patient-specific optimization, high manufacturing costs, and production challenges are major barriers. In silico tools and exosome coatings are expected to accelerate progress [83].

Device coatings are evolving to improve tissue integration, bone regeneration, and angiogenesis, including silver-based antimicrobial and nano-hydroxyapatite coatings [84,85]. Trials are exploring intratumoral superparamagnetic iron oxide nanoparticles (Nanotherm) with alternating magnetic fields (NCT06271421), gold nanoshells with near-infrared therapy (AuroLase; NCT01679470), photoimmunotherapy with antibody-dye conjugates plus light activation (NCT03769506), hafnium oxide radiosensitizers (NBTXR3, approved in Europe as Hensify; NCT02379845), and ocular implants releasing biologics (NCT04576689). Early clinical studies of nanomotors and implantable nanosensors indicate potential for therapeutic micro- and nanosystems (e.g., a nanomotor platform for peri-implantitis is in a phase 0 trial [20]; energy-harvesting nanosensors for device powering are under development [86]). Prototypes combining sensors, power sources, and cameras have been reported [87].

### 9.2. Testing of Toxicity to Living Systems and LCA

Guidelines for the characterization of nanomaterials have been developed [88], which enable researchers to provide a comprehensive physicochemical characterization. With increased knowledge of the influence of these parameters on toxicity, more recent studies—beyond simple cytotoxicity screening—also include nano-specific toxicity data such as uptake, ROS production, and inflammation for the developed nanodrugs, and compare the effect to the payload alone (one example is [89]). Experts request LCAs not only for the use phase but also for production/manufacturing and recycling/reuse [90]. The use of green chemistry is increasing.

### 9.3. Use of In Silico Tools

**Figure 3:**
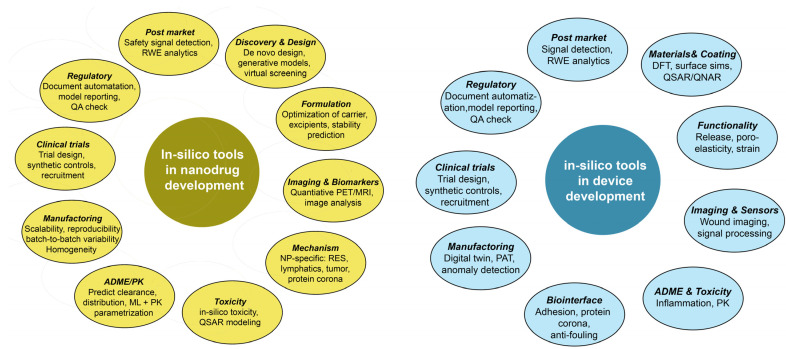
Uses of in silico tools in nanodrug and nano-enabled device development. Abbreviations: ADME, absorption, distribution, metabolism, excretion; DFT, design for testing; ML, machine learning; PAT, process analytical technology; PBPK, physiologically based pharmacokinetic; PK, pharmacokinetics; QA, quality assessment; QSAR, quantitative structure–activity relationship; QNAR, quantitative nanostructure–activity relationship; RES, reticuloendothelial system; RWE, real-world evidence; sims, secondary-ion mass spectrometry.

In silico tools, mechanistic models and AI are expected to increase the number of approved nanodrugs by supporting multiple steps throughout development (Figure 3). Here, AI denotes systems that perform tasks traditionally requiring human intelligence. Machine learning (ML) is a subfield of AI that employs statistical models to analyze large datasets and discover patterns. Within ML, deep learning (DL) is used far more often in nanodrug development than reinforcement learning (RL) [91]. Nevertheless, RL may prove useful for certain device applications, such as implant control, smart drug-delivery systems, or the navigation of nanorobots [92].

Beyond materials identification, in silico tools may be useful in controlled manufacturing, safety and toxicity testing, ADME (absorption, distribution, metabolism, excretion) studies, clinical-trial design, and regulatory-submission support during later development phases [93,94].

The most established applications for nanodrugs are in nanocarrier design, e.g., prediction of particle size, polydispersity, drug-loading efficiency, agglomeration, and stability [95]. Descriptors allow the prediction of ROS generation, cytokine release, and inflammatory responses, which enables prediction of cytotoxicity, hemocompatibility, and immune activation [62]. Protein corona prediction is increasingly used to predict biodistribution, immunogenicity, and cellular uptake [96]. Various pharmacokinetic/pharmacodynamic (PK/PD) models—including hybrid mechanistic/ML approaches—can predict ADME behavior and inform first-in-human dosing and patient stratification for clinical trials [97]. Using existing databases, toxicity predictions are feasible and can help prioritize safety studies and reduce animal use. However, programs for the prediction of PK and biodistribution of nanomaterials are not yet mature because too few high-quality in vivo datasets are available. Multi-endpoint predictions and in vivo behavior forecasts for nanomedicines are available through both open-source and commercial software [98]. Although use in industrial scale-up is highly relevant, AI is currently used sparingly for this application. Examples of the use of AI in material identification, optimization, and biodistribution/tumor targeting of nanodrugs include the optimization of the lipid composition of nanocarriers for mRNA delivery systems by ML [99], the discovery of a new polymer that successfully delivered siRNA to lung cells in vivo [100], and the identification of critical parameters for the biodistribution of doxorubicin-loaded nanoparticles [101].

In silico tools also support the optimization of manufacturing, quality control, safety assessment, and regulatory support for nano-enabled devices. ML is particularly valuable for identification of optimal composition for the desired properties, process monitoring and optimization (improving coating functionality and mechanical strength), and sensor integration [91,102]. It also helps in signal interpretation and signal enhancement [103]. AI use in the control of nanorobots is promising but, currently, only early prototypes are available. AI is also used in wearable devices [104]. In contrast to nanodrugs, the use of AI in industrial nanofabrication and quality control is widespread [105]. Concrete examples of improved nano-enabled devices include designing geometries that yield desired electromagnetic responses for nanophotonics [106], manufacturing controls that improve yield and reproducibility of nanoscale features [107], and better identification of disease-specific signatures [108].

## 10. Conclusions

The initially proposed multifunctional nanoparticles and nanorobots have proven to be harder to realize than early expectations suggested. Over the past decade and a half, experience has focused objectives and refined design strategies. Future goals for nanodrugs include programmable nanosystems that deliver gene editors safely and transiently to specific cell types, enabling curative genetic therapies for diseases. Smart nanoparticles that sense intracellular states will improve cellular targeting and reduce off-target effects. Greater use of biodegradable carriers to avoid long-term persistence and environmental accumulation is expected. Ideally, the nanocarrier should be fully degraded immediately after the payload has been released. More nanodrugs are expected to be developed for oral administration. For nano-enabled devices, priorities include improved tissue integration (vascularized scaffolds, neuronal interfaces) and regenerative support, whereas implantable therapeutic networks based on nanosensors remain a longer-term vision.

Nanotoxicity testing of nano-based medical products has increased the focus on changes in particle parameters in physiological fluids and on assessing uptake in different cell types. Mode-of-action studies should include lysosomal function, ROS generation, pro-inflammatory activity, complement activation, genotoxicity, and barrier permeation in vitro. In vivo studies should look for chronic inflammation, accumulation in RES organs, and CARPA.

LCA should be performed not only with a focus on the use phase but should also include production and disposal. It is expected that green synthesis—where key parameters for the synthesis of Au, Ag, ZnO, iron oxide, Cu, Pd, and CuO nanoparticles are known—will have a positive impact [109].

Increased use of AI is expected for predicting protein corona formation, for industrial applications in nanodrug manufacturing, and for the control of biomedical robots for nano-enabled devices. The currently low use of AI in LCA and in predicting clinical outcomes is expected to increase. Reliable AI use depends on robust platforms and curated data repositories; resources such as NanoCommons, caNanoLab, Nanomaterial Registry, Nano-SolveIT, and the FDA Predictive Toxicology Roadmap will help develop the technology further [110].

## Figures and Tables

**Table 1:** Comparison of ideal and FDA-approved nanodrugs. Parameters achieved in FDA-approved products are indicated in green, and missing parameters in red. Abbreviation: EPR, enhanced permeability and retention; PEG, polyethylene glycol.

Ideal Feature	FDA-Approved Reality
Nanodrugs	
Targeted cellular uptake	Some passive (EPR); active ligands rare
pH, light, enzyme, or magnetic field-triggered release	Mostly absent
Multifunctional theranostics	Separate systems; some imaging agents
Biodegradability + clearance	Present for lipids/polymers but clearance varies
Immune system evasion	PEGylation; still some immunogenicity
Personalized/adaptable payload	Rare
Nano-enabled Devices	
Active sensing/feedback	Mostly absent
Smart/triggered response	Limited to passive material properties
Antimicrobial & surface function	Nanocoatings improve antimicrobial & healing surfaces
Enhanced tissue integration	Nanostructured surfaces on implants
Multifunctional theranostics	Devices are single purpose
Real-time patient data output	Mostly none
Miniaturized implantable sensors	Only nanoscale coatings

**Table 2:** Nano-based products used in the clinic. Years in brackets indicate the year of approval by the FDA. Abbreviation: AB, antibody; ATTR, Transthyretin amyloidosis; PEG, polyethylene glycol.

Drug	Description	Indication
Clearblue pregnancy test (1988)	Colloidal gold particles	Pregnancy
Adagen (1990)	PEG-conjugated adenosine deaminase	Immunodeficiency
Oncaspar (1994)	PEG-conjugated L-asparaginase	Oncology
Doxil (1995)	PEGylated liposomal doxorubicin	Oncology
DaunoXome (1998)	Liposomal daunorubicin	Oncology
AmBisome (1997)	Liposome encapsulated amphotericin B	Fungal infection
DepoCyt (1999)	Liposomal cytarabine	Oncology
Mylotarg (2000)	Anti-CD33 AB-ozogamicin conjugate	Oncology
Rapamune (2000)	Nanocrystalline sirolimus	Graft rejection
Genexol-PM (2000)	Polymeric micelle paclitaxel	Oncology
Visudyne (2000)	Liposomal Photosensitizer	Age-related macular degeneration
PegIntron (2001)	PEG-conjugated interferon alpha	Infection
Eligard (2002)	Leuprorelinacetat + Poly(DL-lactid-co-glycolid)	Oncology
Zevalin (2002)	Radio-immunoconjugate	Oncology
Oral nanocrystal formulations (since 2003)	Emend, TriCor, Megace ES, Triglide, Focalin XR, Ritalin LA, Invega, Naprelan, Ryanodex, Zanaflex	Nausea, hypercholestolemia, anorexia, behavioral disorders, pain, spasticity
Estrasorb (2003)	Micellar 17-beta estradiol	Menopausal symptoms
Somavert (2003)	PEG-conjugated growth hormone analog	Acromegaly
Macugen (2004)	PEG-conjugated RNA-Aptamer	Age-related macular degeneration
Abraxane (2005)	Albumin-bound paclitaxel	Oncology
Acticoat/Aquacel-Ag (2007)	Silver-containing coatings	Wound care
Invega Sustenna/Trinza (2009)	Long-acting nanocrystals	Schizophrenia
Ferumoxytol (2009)	Iron oxide nanoparticles	Anemia/ Imaging
Feraheme (2009)	Colloidal iron oxide	Anemia
Silverline, silver Soaker (since 2010)	Silver coating	Orthopedics, dentistry, surgery, cardiovascular diseases
Sensors (since 2010)	Gold or magnetic nanoparticles	Point of care
Krystexxa (2010)	PEG-conjugated uricase	Refractory gout
Adcetris (2011)	Anti-CD30 AB-auristatin conjugate	Oncology
Marqibo (2012)	Liposomal vincristine	Oncology
Kadcyla (2013)	Anti-HER2 AB-maytansinoid conjugate	Oncology
nanoLOCK (2014)	Nano-textured surface	Orthopedics
Plegridy (2014)	PEG-conjugated Interferon beta	Multiple sclerosis
Adynovate (2015)	PEG-conjugated coagulation factor VIII	Hemophilia A
Onivyde (2015)	Liposomal irinotecan	Oncology
Aristada (2015)	Nanocrystalline prodrug	Schizophrenia
Lipusu (2016)	Liposomal paclitaxel	Oncology
Vyxeos (2017)	Daunorubicin/cytarabine in liposomes	Oncology
Zilretta (2017)	Triamcinolone acetonide polymeric nanogel	Osteoarthritis
Rebinyn (2017)	PEG-conjugated coagulation factor IX	Hemophilia B
Besponsa (2017)	Anti-CD22 AB-calicheamicin conjugate	Oncology
Onpattro (2018)	siRNA in lipid nanoparticles	Hereditary ATTR
Mircera (2018)	PEG-conjugated Epoetin beta	Anemia
COVID-19 vaccines (2020)	mRNA in lipid nanoparticles	COVID-19
Polivy (2019)	Anti-CD79b AB-auristatin conjugate	Oncology
Enhertu (2019)	Anti-HER2 AB-deruxtecan conjugate	Oncology
Myocet (2020)	Non-PEGylated doxorubicin	Oncology
Trodelvy (2020)	Anti-trop2 AB-SN-38 conjugate	Oncology
Apretude (2021)	Long-acting nanocrystals	HIV
Cabenuva (2021)	Nanocrystalline cabotegravir + rilpivirine	HIV

**Table 3:** Specific properties of nano-based products and consequences of toxicity testing.

Nano-Specific Property	Action
Influence of size, shape, surface charge, surface properties on toxicity	Reproducible, homogenous production, assessment of uptake in different cell types
Formation of protein corona	Testing of particles absorbed in relevant physiological solution, determination of protein corona
Generation of oxidative stress, membrane damage, lysosomal damage, ion release, inflammation, complement activation, genotoxicity)	Determination of ROS generation and measurement of ion release; inclusion of cellular assays for nano-specific toxicity
Restricted access to cells or tissues and translocation	Exposure-specific models, e.g., co-cultures and reconstructed tissues, evaluation of permeation across epithelial barriers
Accumulation and chronic toxicity	Long-term exposure, study of RES organs, inflammation markers, fibrosis and granuloma formation
Interference with conventional screening assays	Inclusion of interference controls, e.g., for color, fluorescence, dye metabolization

**Table 4:** Differences in relevant LCA parameters between nanodrugs and nano-enabled devices.

Parameter	Nanodrug	Nano-Enabled Device
Functional unit	Dose- and formulation-dependent	Assessment is performance-driven and durability-based
Life cycle stages	Synthesis of drug and nanocarrier, formulation, sterilization, packaging, cold chain, excretion and wastewater treatment	Raw material extraction, manufacturing and assembly, energy consumption and wear during use, recycling, incineration
Production	Batch variability, low volume, high-purity synthesis	Scale-up manufacturing, nanomaterials yields and losses
Exposure pathways	Patient, patients’ excretions, hospital effluents	Manufacturing, abrasion, wear, disposal or recycling
Impact	Human toxicity and ecotoxicity	Climate change, resource depletion, human toxicity
End-of-life	Metabolism, excretion, degradation, persistence in wastewater	Recycling, recovery of materials, release during shedding or incineration

## Data Availability

No new data were created or analyzed in this study. This article is a literature review, and all information is derived from previously published sources.

## Abbreviations

The following abbreviations are used in this manuscript:

ADME	Absorption, distribution, metabolism, excretion
AI	Artificial intelligence
ATTR	Transthyretin amyloidosis
CARPA	Complement activation-related pseudoallergy
COVID-19	Coronavirus disease 2019
DL	Deep learning
EPR	Enhanced permeability and retention
FDA	Food and drug administration agency
GMP	Good manufacturing practice
GPS	Global Positioning System
HER-2	Human epidermal growth factor receptor 2
HIV	Human immunodeficiency virus
IM	Intramuscular
IV	Intravenous
LCA	Life cycle assessment
ML	Machine learning
MPS	Mononuclear phagocyte system
PAT	Process analytical technology
PBPK	Physiologically based pharmacokinetic
PEG	Polyethylene glycol
PK/PD	Pharmacokinetic/pharmacodynamics
PSMA	Prostate specific membrane antigen
QSAR	Quantitative structure-activity relationship
QNAR	Quantitative nanostructure-activity relationship
RES	Reticuloendothelial system
RL	Reinforcement learning
ROS	Reactive oxygen species
RWE	Real world evidence
SC	Subcutaneous
